# NAVIGATING DISASTERS: A CASE STUDY VA AND NON-VA HOME-BASED LONG-TERM CARE IN PUERTO RICO FOLLOWING HURRICANE MARIA

**DOI:** 10.1093/geroni/igz038.1022

**Published:** 2019-11-08

**Authors:** Leah M Haverhals, Deisy Hernandez-Lujan

**Affiliations:** 1 Seattle-Denver Center of Innovation, Denver, Colorado, United States; 2 Seattle-Denver Center of Innovation, Aurora, Colorado, United States

## Abstract

This research describes how Department of Veterans Affairs (VA) and non-VA home-based long-term care (LTC) environments prepared for and secured the safety and wellbeing of elderly and disabled persons in the wake of Hurricane Maria, which struck Puerto Rico on September 20, 2017. In-person interviews, home visits, and field observations were conducted in Puerto Rico from January-March 2019. Staff from three of the VA’s Caribbean Healthcare System’s home-based LTC programs were interviewed, as well as caregivers in non-VA LTC environments. Veterans, family members of Veterans, family members of VA caregivers, and community members with expertise in disaster recovery were also interviewed for a total of N = 58 interviews and N = 12 home visits. Preliminary results of qualitative content analysis show VA and non-VA LTC environments prepared residents and caregivers for Hurricane Maria through providing education and recommendations in advance, including having enough medications and food on hand. Participants described Hurricane Maria not simply as a disaster but a “crisis” and a storm unlike any they had ever experienced. The interconnected nature of the VA seemed to provide a stronger support network compared to non-VA environments that were often independently run. Health of Veterans and non-Veterans was reported to be mostly stable during recovery. Perspectives from VA and non-VA entities allowed for a fuller picture to emerge around how Hurricane Maria impacted home-based LTC environments in Puerto Rico. This research can inform policies and procedures for such environments caring for elderly and disabled populations in areas prone to disasters.

